# Illustrated operative management of spontaneous bleeding and compartment syndrome of the lower extremity in a patient with acquired hemophilia A: a case report

**DOI:** 10.1186/1752-1947-8-132

**Published:** 2014-04-30

**Authors:** Thorsten Jentzsch, Brigitte Brand-Staufer, Frank P Schäfer, Guido A Wanner, Hans-Peter Simmen

**Affiliations:** 1 Division of Trauma Surgery, Department of Surgery, University Hospital Zürich, Rämistrasse 100, 8091 Zürich, Switzerland; 2 Division of Hematology, Department of Internal Medicine, University Hospital Zürich, Zürich, Switzerland

**Keywords:** Acquired hemophilia A, Compartment syndrome, Factor VIII deficiency, Fasciotomy, Lower extremity, Operative management

## Abstract

**Introduction:**

Spontaneous bleeding resulting in compartment syndrome at the lower adult leg due to acquired hemophilia A is rare. There are no reports on operative management of this entity.

**Case presentation:**

We present a case of atraumatic compartment syndrome of the lower leg due to acquired factor VIII deficiency, in an 83-year-old Caucasian man of European descent. He was treated surgically with a long and complicated postoperative course after presenting to a community hospital with a 2-day history of increasing pain and swelling in his left lower leg without a previous history of trauma.

**Conclusions:**

Awareness, prompt diagnosis and effective treatment of compartment syndrome caused by a rare bleeding disorder, which is usually acquired by the elderly, is essential and may spare a patient from surgery or even limb loss, if early administration of recombinant factor VIIa is effective. The course of disease in a patient with operative management of spontaneous bleeding, compartment syndrome and acquired hemophilia A may be prolonged. However, an interdisciplinary approach with meticulous surgical treatment and bleeding management with recombinant factor VIIa as well as inhibitor eradication by immunosuppressive treatment can be successful and expensive.

## Introduction

Spontaneous bleeding and compartment syndrome due to acquired hemophilia A, which is defined by factor VIII deficiency caused by an inhibitory antibody to factor VIII, is extremely rare [1]. This is in contrast to the more well-known and rather common compartment syndrome due to traumatic etiologies, of which the majority may be attributed to fractures [2]. Only a few case reports have focused on compartment syndrome in the realm of acquired hemophilia A in the adult [3-5] and pediatric population [6-11], respectively. Almost all of these case reports addressed problems in the upper extremities [4-7] or described conservative treatment [3,5]. There is a lack of studies [6,7] on operative management of compartment syndrome due to acquired hemophilia A and none describe this entity on the lower extremities. Therefore, we present a case of atraumatic compartment syndrome of the lower leg due to acquired hemophilia A, in an elderly Caucasian man who was treated surgically with a long and complicated postoperative course.

## Case presentation

An 83-year-old Caucasian man of European descent presented to a community hospital with a 2-day history of increasing pain and swelling in his left lower leg without a previous history of trauma. His relevant past medical history included fasciotomy of an atraumatic compartment syndrome of his left thigh 2 months ago, traumatic hemarthrosis of his right knee half a year ago and a 2-year course of low-dose steroid therapy for polymyalgia. Furthermore, he had hypertensive cardiomyopathy, permanent atrial fibrillation, benign prostate hyperplasia, presbyacusis, and a history of surgery for patellar tendon rupture 1 year ago. Aside from prednisolone 5mg/day, he took nebivolol 2.5mg/day, torasemide 10mg/day and tamsulosin 0.4mg/day. Warfarin had been stopped 2 months ago after his compartment syndrome of the thigh.

During physical examination, he had pain at rest, to palpation and with passive stretching of the foot (Numeric Rating Scale 4 to 5) at his left lower leg. Marked swelling with emerging tension blisters was found at his proximal anterior tibia. His peripheral pulses were palpable and his neurologic status was intact. There were no systemic signs of disease. Ultrasound revealed a hematoma, 6×3×2cm, in the anterior tibial compartment, but no evidence of vessel pathology or deep vein thrombosis. Compartment pressure of the anterior tibial compartment was measured with the help of a pressure monitoring device and was markedly elevated. Laboratory findings included an elevated C-reactive protein of 94mg/L (normal <5mg/L), decreased hemoglobin of 86g/L (normal 125 to 172g/L), decreased hematocrit of 0.28L/L (normal 0.37 to 0.49L/L) and prothrombin time (PT) of 87% (normal 70 to 100%). Activated partial thromboplastin time (aPTT; normal 24 to 36 seconds) was not measured because it was falsely not deemed relevant or simply forgotten at that time.

The diagnosis of compartment syndrome was made and the patient underwent immediate lateral fasciotomy with a limited incision of approximately 5cm of the anterior tibial compartment by a rather small skin incision and subcutaneous fascia release as described by Mubarak and Owen [12] (Table 1 outlines the course of disease). The anterior tibialis muscles protruded instantly and were still vital. The wound was left open and covered with dressings. Two days later, secondary hemorrhage and foot drop (M3, active movement against gravity) developed. Thus, he was taken to the operating room again and a hematoma within the anterior tibial compartment was evacuated. Postoperatively, his foot drop mildly improved. Again, 2 days later, another secondary hematoma occurred and he was transferred to a larger regional hospital in order to treat his condition, with its peculiar laboratory findings and uncontrollable bleeding, in the realm of an interdisciplinary approach. The lateral fasciotomy wound was lengthened [13]. Furthermore, his aPTT was measured and found to be elevated, which prompted further hematologic evaluation. On the fifth postoperative day, diagnosis of acquired hemophilia A was finally made by a hematology consultant. Due to massive unstoppable bleeding of approximately 0.5L/hour accompanied by a fall in hematocrit from 29% to 19.5%, despite the administration of four units of red blood cells, recombinant factor VIIa (rFVIIa) was used as a single bleeding control agent and corticosteroids (1mg/kg body weight) were increased for inhibitor eradication. Of note, no other blood products or factor replacements were administered. In our hospital, we also do not administer factor VIII inhibitor bypass activity (FEIBA) routinely in acquired hemophilia A, but reserve this treatment for refractory hereditary cases. With this drug therapy in place and the assumption that his uncontrolled bleeding had been controlled, vacuum-assisted closure (VAC) therapy was instigated. VAC therapy represents the regional standard therapy for most open wounds and even though it may monitor the amount of bleeding, it does not provide visual control. However, secondary hemorrhage occurred again and the patient was transferred to a university hospital.

**Table 1:** Course of disease

**Day(s) after diagnosis**	**Clinical symptoms**	**Laboratory (factor VIII, Bethesda test, activated partial thromboplastin time)**	**Therapy**
**0**	Compartment syndrome		Minimally invasive lateral fasciotomy
**2**	Secondary hemorrhage and foot drop (M3, active movement against gravity)		Hematoma evacuation
**4**	Secondary hemorrhage	Recognition of prolonged activated partial thromboplastin time	Lateral fasciotomy elongation
**5**	Diagnosis of acquired hemophilia A		Recombinant factor VIIa and corticosteroids
**6, 7**		3%, 3 Bethesda units, 76 seconds	Vacuum-assisted closure (change)
**9, 11**	Secondary hemorrhage		Polyvinyl alcohol foam, jetting hose and absorbable dressing
**15**	Secondary hemorrhage	3%, 8 Bethesda units, 40 seconds	Vacuum-assisted closure polyvinyl alcohol foam, jetting hose and absorbable dressing
**17–28**		9–33%, 4 Bethesda units, 26–35 seconds	Polyvinyl alcohol foam, jetting hose and absorbable dressing changes
**31**		41%	Vacuum-assisted closure
**35**		normal	Vacuum-assisted closure change
**43**		normal	Split-thickness skin graft
**46–106**	Delayed wound healing	normal	Open wound therapy with AQUACEL® and Mepitel®
**107**		normal	Second split-thickness skin graft
**108–170**	Healed wound	normal	Dismission from traumatologic treatment

**Figure 1:**
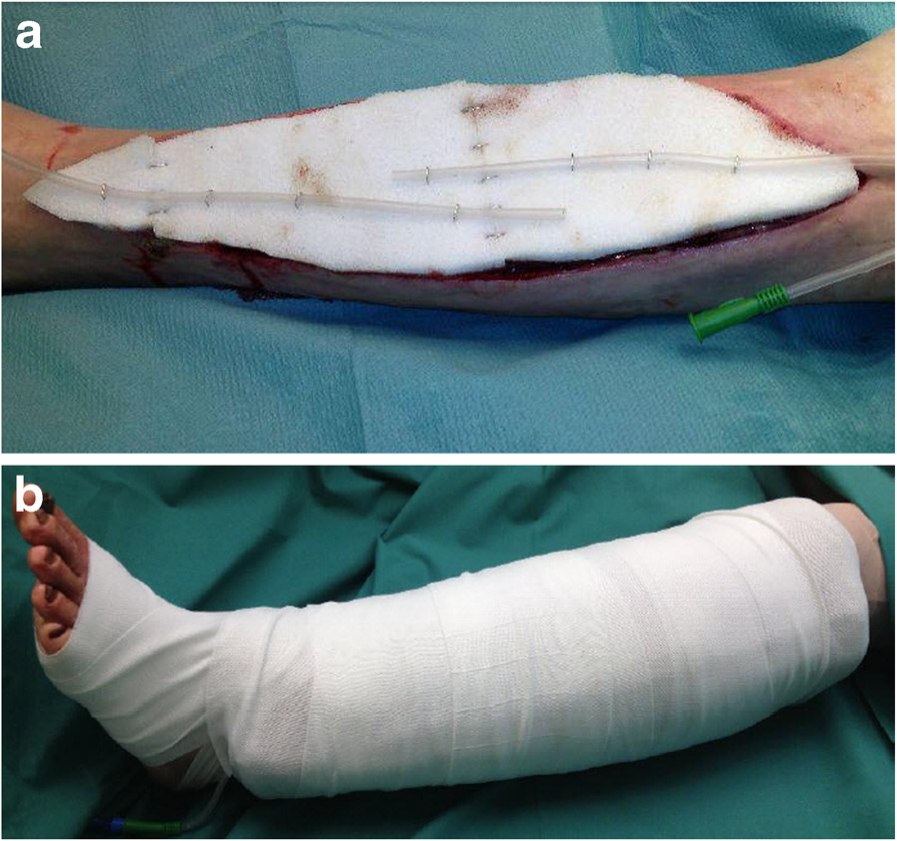
**Special wound dressings. a**. Wound dressing on lower leg with PVA foam and jetting hose. **b**. Bulky absorbable wound dressing on lower leg.

**Figure 2:**
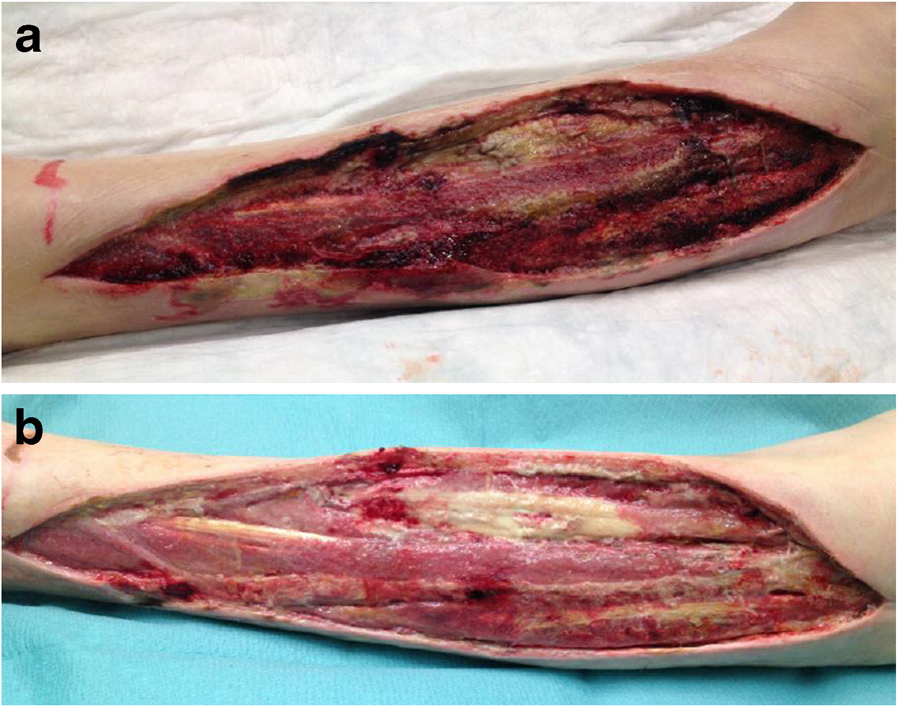
**Uncontrollable bleeding. a**. Wound with uncontrollable bleeding on lower leg 2 weeks after diagnosis. **b**. Wound with controlled bleeding on lower leg 1 month after diagnosis.

**Figure 3:**
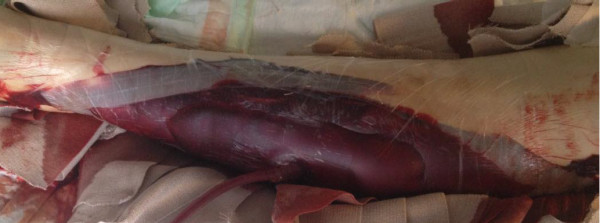
Secondary hemorrhage with vacuum-assisted closure therapy on lower leg.

**Figure 4:**
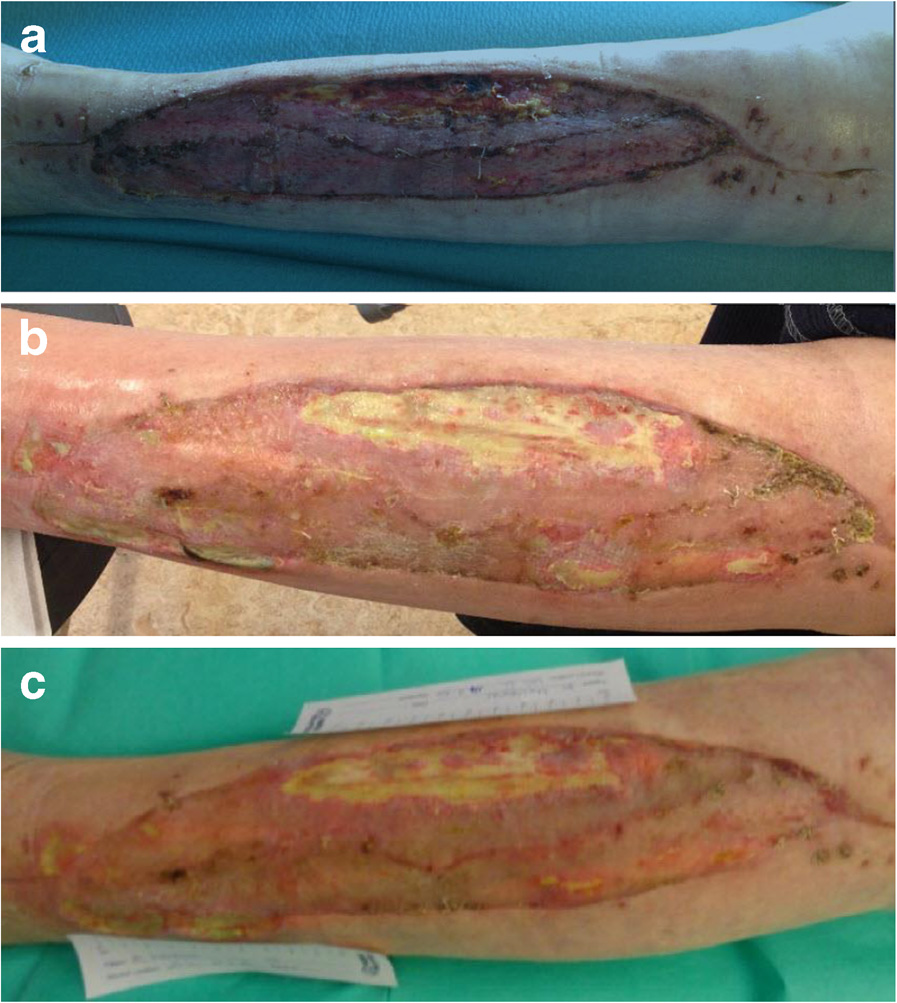
**Delayed wound healing. a**. Delayed wound healing in the anterior part of the split-skin graft on the lower leg 2 weeks after split-skin grafting and 2 months after diagnosis. **b**. Wound on lower leg 1.5 months after split-skin grafting and 3 months after diagnosis. **c**. Wound on lower leg 2 months after split-skin grafting and 3.5 months after diagnosis.

**Figure 5:**
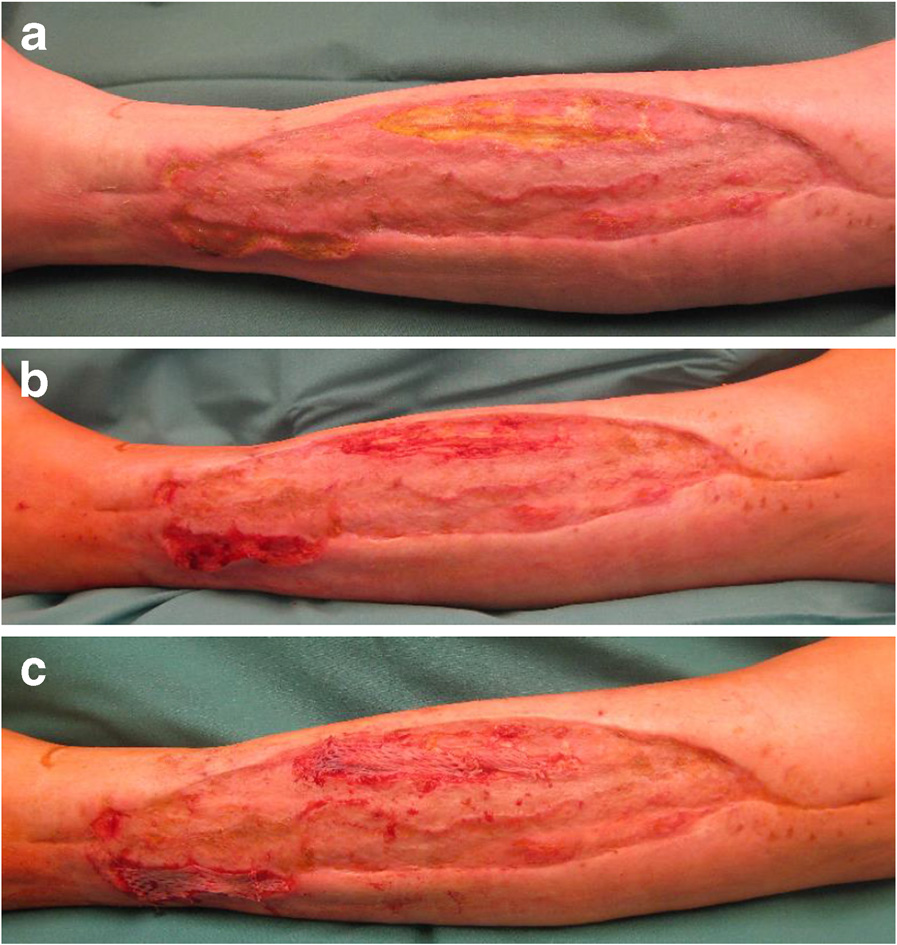
**Second split-thickness skin graft. a**. Delayed wound healing after 3.5 months. **b**. Intraoperative wound after debridement after 3.5 months. **c**. Second split-thickness skin graft after 3.5 months.

**Figure 6:**
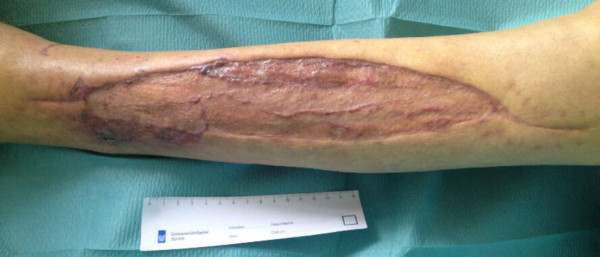
Healed wound on lower leg after 5 months.

In order to halt the uncontrollable bleeding, the wound was covered with a special dressing with polyvinyl alcohol (PVA) foam, a jetting hose through which the wound was kept moist and a big bulky absorbable dressing (Figures 1a, 1b). On day 15, implementation of VAC therapy failed again and uncontrollable bleeding led to further therapy with the special dressing mentioned above (Figures 2a and 3). By this time, the patient had received at least seven units of red blood cells and almost 20 administrations of NovoSeven® (rFVIIa), usually in the dose of 7mg each. No other blood products were used except for 1g of tranexamic acid intraoperatively on his arrival at the university hospital. At that point his factor VIII was still 3%. For the next 2 weeks, dressing changes were undertaken at regular intervals of approximately 3 days. Administration of rFVIIa at regular intervals, at least once a day just shortly before dressing changes was continued. His factor VIII started to rise slowly, beginning on day 17 with 9%. After 4 weeks, his factor VIII had risen to 41% and VAC therapy could be applied again in order to condition the wound for definite closure (Figure 2b). After another VAC change, and rise of factor VIII to 88%, a split-thickness skin graft was carried out 1.5 months after the first fasciotomy. At this time, rFVIIa support was no longer necessary. After leaving a VAC on top of the split-thickness skin graft for 5 days, mild delayed wound healing was observed in the anterior middle and posterior distal parts of the wound (Figure 4a). This was treated with AQUACEL® (primary wound dressing) and Mepitel® (wound contact layer), (wound contact layer), while the rest of the wound was dressed with dry padding. In the meantime, factor VIII had increased >100% and corticosteroid therapy could be reduced in a stepwise fashion approximately twice a month. After discharging the patient into a rehabilitation clinic 2 months postoperatively, he was frequently seen in our out-patient clinic (Figure 4b). After 3.5 months, small parts of the wound had still not healed (Figure 4c), and a split-thickness skin graft was carried out for a second time (Figure 5a to 5c) Afterward, wound healing went slowly, but finally succeeded approximately 5 months after his initial presentation while his factor VIII remained normal and prednisone was stopped 5 months after diagnosis (Figure 6). He had 5 out of 5 muscle strength globally, but needed to ambulate with crutches after being confined to bed rest for a long time.

Unfortunately, the patient was hospitalized again after 4.5 months. This time, he had developed a spontaneous hematoma in his left iliopsoas muscle. It was not due to a relapse of acquired anti-factor VIII deficiency, but rivaroxaban, which had been administered for intermittent atrial fibrillation. From that point, oral anticoagulants were stopped and he only received low-molecular-weight heparins daily while immobilized. Unfortunately, he developed cholangitis, which was suspected due to icterus and increased cholestasis/liver parameters followed by an ultrasound and a computed tomography scan of the abdomen as well as a magnetic resonance cholangiopancreatography and finally confirmed by a liver biopsy. He is scheduled for a rehabilitation program in order to regain normal ambulation without crutches upon his discharge from our hospital.

## Discussion

Acquired hemophilia is a rare hemorrhagic disorder and occurs at a rate of approximately one person per million each year [8,14-19]. It mainly affects the elderly without gender preference. Spontaneous remission may occur in up to one-third of patients [20]. Nonetheless, the mortality rate has been reported to be as high as 22% in a survey by Green and Lechner [8]. Acquired hemophilia is caused by formation of an inhibitory antibody directed against coagulation factor VIII. Its origin is idiopathic in approximately half of cases (46 to 65%), but may be related to other medical conditions, such as autoimmune disorders in about 18%, malignancies (7 to 15%) or the post-partum period (7 to 11%) [15,21]. It may lead to severe and potentially life-threatening bleeding once factor VIII decreases <25%. Suspicion usually arises with uncontrollable bleeding after a surgical procedure or spontaneous bleeding in patients with no history of a bleeding disorder [22]. The diagnosis should be suspected with prolonged aPTT. A prolonged aPTT may be attributable to several causes, such as coagulation factor deficiencies, lupus anticoagulant or heparin therapy and differentiation is achieved by mixing tests, where the plasma of the patient is mixed with normal plasma [23]. Initially normal, but increased aPTT after 2 hours of incubation is suggestive of autoantibodies to factor VIII. Yet, the PT remains normal. Mixing studies of aPTT can be performed in most laboratories and support the suspicion of acquired hemophilia. More specifically, factor VIII can be quantified and is usually decreased <50%. Afterward, the antibody is assessed with the Bethesda assay, which measures residual factor VIII in the plasma of the patient after incubation with plasma from a healthy person [24,25]. Therapeutic approaches include bleeding control with a so-called bypassing agent, either rFVIIa (NovoSeven®) or activated prothrombin complex (FEIBA®) and antibody eradication with prednisone alone or in combination with other immunosuppressives, such as cyclophosphamide or rituximab. RFVIIa activates factor X via the extrinsic system in the absence of factor VIII. Thus, thrombin and fibrin are activated and blood clotting follows [26]. However, rFVIIa has a short half-life of only 2 hours and requires frequent administrations [27]. Prednisone is the classic first-line therapy for antibody eradication and is effective in about 30 to 75% of cases [14]. Cyclophosphamide has been shown to increase the overall response rate, but is usually not used initially due to its severe side effects, particularly infectious complications in the cohort of old to very old patients. In addition, transfusions are necessary in almost 90% of cases.

Compartment syndrome is rare with an incidence of three per 100,000 people [2]. Men are more commonly affected. The origin is a rise in pressure inside a muscle compartment [28]. Compartment syndrome is often caused by hematoma formation due to fractures. There are four muscle compartments in the calf. The anterior compartment is most commonly affected, it contains the dorsiflexors of the foot and the deep peroneal nerve; the lateral compartment contains the foot elevators, part of the plantar flexors and the superficial fibular nerve. The posterior compartment is subdivided into a superficial and deep compartment; it contains the plantar flexors and the tibial nerve. Failure to recognize compartment syndrome or delay in treatment may lead to serious disability. Compartment syndrome is a clinical diagnosis. Progressive nerve symptoms, such as paresthesia, are the first symptoms and usually arise within 30 minutes. The remaining classically described “six Ps” – pain, pallor, pulselessness, pressure, poikilothermia and paralysis – often arise too late, because irreversible tissue damage may begin within 3 hours, as described in a cohort analysis of 76 patients by Vaillancourt *et al*. [29]. Compartment pressures can be measured non-invasively or invasively. Pressures >30mmHg have been accepted as an indication for fasciotomy in order to avoid irreversible tissue damage [30]. Fasciotomy incisions should be large enough to sufficiently decompress affected compartments and in the case of the calf measure about 15cm. The approach is either through a single lateral parafibular incision or combined anterolateral and posteromedial incisions; the latter is more common. Skin closure should not be performed until the swelling has reached its peak. As described in a review by Shadgan *et al*. [31], there are several options, such as primary closure with skin reapproximation, split-thickness skin grafting, dermatotraction with staples, vessel loops and vacuum-assisted wound closure. We usually use vacuum-assisted wound closure for wound conditioning before split-thickness skin grafting. As summarized by Holle *et al*. [32], it functions by stabilizing tissue, giving protection against infections, improving blood circulation within tissues, and induction of wound healing. McCallon *et al*. [33] reported that vacuum-assisted wound closure was able to decrease the volume of the wound better than normal wound dressings (28 versus 10%) and also led to faster wound closure (23 versus 43 days). In our case, vacuum-assisted wound closure failed in the initial phase because of the uncontrollable bleeding, but was effective once bleeding was controlled. As mentioned by Ojike *et al*. [34], a split-thickness skin graft is usually used to cover large wounds after compartment syndrome. Schneider *et al*. [35] attributed wound healing to the equal pressure distribution on the skin graft in 98% of a total of 100 patients, who were treated with vacuum-assisted wound closure on top of the skin graft for a couple of days.

From an economic standpoint, rFVIIa may be expensive for health care providers since a single dose for an average adult patient costs approximately US$7000. Due to the short half-life, there is always a need for more than one administration daily for a course of a couple of weeks. Thus, expenses up to a hundred thousand may arise quickly. In our case, rFVIIa was given 49 times, which added up to approximately $340,000. However, the hospital received approximately $60,000 for the diagnosis-related group. Thus, there is a deficit of $280,000, which has to be paid by the treating hospital. However, these costs are comparable to other drugs for rare diseases, such as $250,000 a year for lomitapide for homozygous familial hypercholesterolemia or $295,000 per year for teduglutide for short bowel syndrome [36]. A new drug, ibrutinib, for chronic lymphatic leukemia will also cost $130,000 per year [37]. Therefore, it is reasonable to transfer these patients with rare and difficult-to-treat medical conditions to hospitals with a higher level of care. Importantly, treatment with rFVIIa needs to be performed adequately and, as in our case, only be initiated once other factors have been substituted. It is important to point out that the two treatment priorities of acquired hemophilia A are to arrest the acute bleeding with rFVIIa and to eradicate the factor VIII autoantibody with corticosteroids [38].

Our case report points out several important facts. We agree with Ilyas *et al*. [4], who mentioned that atraumatic compartment syndromes due to bleeding disorders deserve special attention and are easily overlooked. Early suspicion, investigation and confirmation of an acquired bleeding disorder are very important in order to avoid invasive investigations or surgeries without adequate support of coagulation. Usually, compartment syndrome is a surgical emergency because irreversible tissue injury begins approximately 6 hours after onset and may lead to tissue necrosis, functional impairment and renal failure [39]. It is only within this very short time window that it may be feasible to perform serial measurements of compartment pressure and postpone surgery in patients with elevated compartment pressures and hemophilia A because uncontrollable bleeding may arise during surgery, especially with factor VIII levels <50% [39]. During the first 6 hours, first-line treatment consists of bleeding control with bypassing agents depending on the underlying acquired disorder. But if compartment pressures remain elevated ≥30mmHg and do not normalize within 2 hours, fasciotomy is needed [40]. Unfortunately, diagnosis of compartment syndrome in the absence of trauma is commonly delayed and made after 6 hours of onset, therefore requiring immediate fasciotomy [11]. Once surgical management is instigated, the course of disease in a patient with spontaneous bleeding, compartment syndrome and acquired hemophilia A may be prolonged, complicated, costly and requires a close collaboration between surgeons and hematologists.

## Conclusions

Awareness, prompt diagnosis and effective treatment of compartment syndrome caused by a rare bleeding disorder, which is usually acquired by the elderly, is essential and may spare a patient from surgery or even limb loss, if early administration of rFVIIa is effective. The course of disease in a patient with operative management of spontaneous bleeding, compartment syndrome and acquired hemophilia A may be prolonged. However, an interdisciplinary approach with meticulous surgical treatment and bleeding management with rFVIIa, which proved to be extremely helpful in our patient, especially pre-operatively, as well as inhibitor eradication by immunosuppressive treatment can be successful and expensive.

## Consent

Written informed consent was obtained from the patient for publication of this case report and any accompanying images. A copy of the written consent is available for review by the Editor-in-Chief of this journal.

## Competing interests

The authors declare that they have no competing interests.

## Authors’ contributions

TJ: Conception and design, acquisition of data, analysis and interpretation of data, drafting the manuscript, revising the manuscript. BBS: Revising the manuscript. FPS: Acquisition of data. GAW: Acquisition of data. HPS: Revising the manuscript. All authors read and approved the final manuscript.
